# Metabolic and immune integration in aging and age-related disease

**DOI:** 10.18632/aging.100626

**Published:** 2014-01-06

**Authors:** Rebecca I. Clark, David W. Walker, Marc S. Dionne

**Affiliations:** ^1^ Centre for the Molecular and Cellular Biology of Inflammation and Peter Gorer Department of Immunobiology, King's College London School of Medicine, London SE1 1UL, UK; ^2^ Department of Integrative Biology and Physiology, University of California, Los Angeles, Los Angeles, CA 90095, USA; ^3^ Molecular Biology Institute, University of California, Los Angeles, Los Angeles, CA 90095, USA

Metabolic regulation and immune responses are tightly coupled in numerous contexts. Chronic and acute infections can drive metabolic disruption and lead to wasting of fatty and lean tissues, a phenomenon apparent in many human infections. Furthermore, several metabolic syndromes, particularly obesity and diabetes, have an inflammatory component which is thought to be a driving force behind the pathology associated with these increasingly common diseases [1]. In aging human populations metabolic dysfunction, particularly insulin resistance, and inflammatory disorders become increasingly common; however, the etiology of these age-related diseases remains unclear [2,3]. Identifying the molecular mechanisms that underlie metabolic-immune integration is therefore of critical importance to our understanding of metabolic syndromes, infection-induced pathology and aging.

In *Drosophila*, the metabolic and immune consequences of aging are tightly correlated, as individuals that show increased antimicrobial gene expression also show systemic metabolic dysfunction [4]. The metabolic and immune phenotypes of individuals undergoing normal aging therefore begin to mimic those of animals experiencing infection-induced metabolic patho-physiology. This occurs in the apparent absence of infection, and its proximate cause remains unknown. In recent work, we have identified the transcription factor MEF2 as a critical transcriptional switch between metabolism and immunity [5]. In *Drosophila*, as in vertebrates, chronic or acute infection results in metabolic disruption. In the adult fat body, a structure analogous to the vertebrate liver and adipose tissue, MEF2 exists in two states with distinct physiological functions. Under normal conditions MEF2 phosphorylated at T20 promotes the expression of key enzymes involved in anabolic metabolism. However, in infected animals T20 phosphorylation is lost and MEF2 associates with the TATA-binding protein, and drives normal expression of multiple antimicrobial peptides. MEF2 therefore acts as a switch between distinct transcriptional states.

The integration of anabolic signaling and immune activation in this manner may represent an efficient program allowing an animal to alter energy usage in the face of challenge. Alternatively, it may arise from mechanistic constraints resulting from the need for MEF2 to serve different transcriptional functions under different conditions. In either case, prolonged immune activation forces the animal into metabolic pathophysiology. In the absence of infection, when chronic immune activation is unnecessary and detrimental to the animal, MEF2 is an ideal target for intervention.

In the context of animal aging the identification of a single protein which when appropriately controlled switches the organism toward anabolic metabolism and away from immune activation is of particular interest. It is easy to speculate that modulation of the phosphorylation status of MEF2 in adult *Drosophila* may ameliorate the affects of aging on two major physiological systems, with profound consequences for the aging organism.

Identifying the signaling mechanisms that regulate T20 phosphorylation of MEF2 will be a key step toward elucidating upstream inputs that modulate immune-metabolic integration via MEF2. Pursuing this question will reveal the events leading to immune and metabolic dysfunction, be it in the context of infection, metabolic syndromes, or normal aging. Such insights from the *Drosophila* model may be particularly helpful in narrowing the field of candidate pathways as this work moves into mammalian model systems. Immune and metabolic signaling pathways are highly conserved throughout species; it is therefore sensible to suggest that the molecular mechanisms underlying the integration of these signals may also be conserved across organisms. In mammals, *Mef2* family proteins have roles in hematopoiesis, and in B cell and T cell function. In addition, *Mef2* family proteins are critical regulators of muscle metabolism [6,7]. However, the potential roles of these proteins as direct activators of innate immune responses and regulators of adipose metabolism remains to be explored in mammalian models. Nevertheless, identification of the *Drosophila* MEF2 immune-metabolic switch represents a significant advance in our understanding of the integrated physiology of the organism.
